# Perceived control and immersion in AI chatbot interaction: a psychological distance perspective

**DOI:** 10.3389/fpsyg.2026.1823399

**Published:** 2026-07-14

**Authors:** Mengdi Wang, Yanxia Li, Wenjing Zhang

**Affiliations:** 1 School of Economics and Management, Harbin Institute of Technology Weihai, Weihai, China; 2 School of Economics and Management, Yanshan University, Qinhuangdao, China; 3 School of Management, Northeastern University at Qinhuangdao, Qinhuangdao, China

**Keywords:** AI-chatbots, human–AI interaction, immersion, perceived control, psychological distance

## Abstract

**Introduction:**

This study investigates how perceived control shapes immersion in AI chatbot interaction through psychological distance. Drawing on psychological distance theory, we distinguish perceived interaction control, defined as users’ perceived control over conversational flow, from perceived content control, defined as users’ perceived control over generated content. We further examine how these two forms of perceived control contribute to reducing temporal, spatial, and social psychological distance in AI chatbot interaction.

**Methods:**

Survey data were collected from 338 AI chatbot users and analyzed using partial least squares structural equation modeling (PLS-SEM) and fuzzy-set qualitative comparative analysis (fsQCA). PLS-SEM was used to examine the net effects among perceived control, psychological distance, and immersion, whereas fsQCA was used to identify alternative configurational pathways leading to high immersion.

**Results:**

The results show that perceived interaction control positively predicts perceived content control and contributes to reducing all three dimensions of psychological distance. Perceived content control further strengthens the reduction of temporal, spatial, and social psychological distance. Reduced temporal and social psychological distance are positively associated with immersion, whereas reduced spatial distance has no significant effect. The fsQCA results reveal multiple sufficient pathways to high immersion.

**Discussion:**

These findings extend psychological distance theory to the context of conversational AI and highlight perceived control as a key mechanism underlying immersive chatbot experiences. By distinguishing perceived interaction control from perceived content control, this study provides a more nuanced explanation of how users become immersed in AI chatbot interaction.

## 1 Introduction

Artificial intelligence (AI) chatbots have increasingly become a central interface for everyday human–AI interaction across service, commerce, and well-being contexts. With advances in generative conversational technologies, AI chatbot use is no longer confined to short, transactional exchanges but increasingly involves extended and adaptive dialogues unfolding over multiple conversational turns (Akpan et al., 2025). As a result, interacting with an AI chatbot more closely resembles participation in an ongoing interaction episode rather than the execution of a simple tool-use task. This shift is consequential because users continuously interpret not only the AI chatbot’s responses but also their own role and influence within the interaction, rendering AI chatbot use a psychologically meaningful experience rather than a purely functional one (Łukasik and Gut, 2025).

Reflecting this shift, recent AI chatbot research increasingly conceptualizes user engagement as a function of interaction experience rather than a direct consequence of system performance. Across application contexts, studies consistently suggest that objective accuracy or technical efficiency alone cannot account for users’ engagement with conversational AI. Instead, engagement is commonly examined in relation to whether interactions align with users’ situational needs, expectations, and subjective experiences, indicating that effective AI chatbot use depends less on functional capability in isolation than on experiential fit (Scherr et al., 2025; Yang et al., 2024). This experience-oriented understanding is echoed in service and consumption research showing that users’ evaluations of AI chatbot interactions are shaped by conversational appropriateness, responsiveness, and communication style, which influence satisfaction, trust, and engagement beyond task performance considerations (Cai et al., 2022; Gao et al., 2025). Synthesizing these perspectives, recent reviews characterize the AI chatbot literature as increasingly centered on experiential fit and interaction quality, while also noting that performance-based explanations remain insufficient to fully capture user responses (Castillo and Caruana, 2025). Despite this convergence, existing research has largely focused on documenting experiential correlates of engagement, leaving unresolved how such experiences are translated into deeper psychological involvement during ongoing human–chatbot interaction (Castillo and Caruana, 2025).

To address the need for mechanism-based explanations in AI chatbot research, scholars have increasingly turned to psychological constructs that can connect users’ interaction experiences with their subsequent responses. Among these, psychological distance has become a central explanatory lens, capturing users’ perceived temporal, spatial, and social closeness to an interaction target (Trope and Liberman, 2003). Grounded in construal level theory, this perspective suggests that reduced psychological distance is associated with more concrete mental representations of the interaction target, which in turn facilitate engagement and involvement (Trope et al., 2007). Consistent with this logic, empirical research across human–AI interaction contexts shows that design features and interaction cues can shape user responses by altering perceived psychological distance. For example, anthropomorphic cues in AI assistants have been found to reduce distance and enhance user evaluations (Li and Sung, 2021), while human-like representations in AI chatbot-mediated counseling influence compliance intentions through distance-related processes (Park et al., 2024). Similar patterns have been observed in commercial AI chatbot interactions, where conversational strategies affect purchase intentions partly by shaping users’ perceived closeness to the AI chatbot (Meng et al., 2025). Taken together, this body of work establishes psychological distance as a robust mechanism linking AI chatbot interaction characteristics to relational and behavioral outcomes across contexts.

Building on this evidence, existing AI chatbot studies have most often incorporated psychological distance as an intermediate psychological state linking interaction cues to user outcomes. In this literature, psychological distance has often been examined as a mediating psychological state through which relatively stable AI chatbot design or communication cues, such as representation style or predefined response strategies, influence user outcomes including trust, compliance intention, and purchase intention (Li and Sung, 2021; Meng et al., 2025; Park et al., 2024). This line of work has been important in establishing the explanatory value of psychological distance. However, because psychological distance is usually examined in relation to relatively predefined design or communication cues, existing models offer less insight into how users’ own interaction-proximal experiences are associated with their perceived closeness to AI chatbots in generative conversational exchanges. This issue becomes particularly salient in multi-turn AI chatbot interaction, where users and AI chatbots jointly shape topic direction, conversational pacing, and response relevance across interaction turns. In such settings, felt closeness is unlikely to be fully explained by predefined design or communication cues alone. Thus, while existing research convincingly demonstrates that psychological distance matters and can be influenced by design or communication cues, it provides limited explanation for why users may experience different levels of psychological closeness and immersive engagement across generative AI chatbot interactions (Castillo and Caruana, 2025).

Insights from human–AI interaction research suggest that this issue may be better understood by paying closer attention to users’ perceived control and sense of agency during interaction. Contemporary theorizing highlights that a central psychological challenge in advanced AI systems lies in balancing increasing machine autonomy with the preservation of human agency, particularly in interactive settings (Sundar, 2020). From this perspective, user experience depends not only on what an intelligent system does, but also on whether users feel able to meaningfully steer the interaction process in accordance with their intentions. Research on the sense of agency further conceptualizes agency as perceived control over one’s actions and their consequences, underscoring its relevance for understanding engagement with proactive and autonomous systems (Legaspi et al., 2024). In conversational AI contexts, this issue becomes especially relevant because users do not simply receive system outputs; rather, they continuously provide prompts, corrections, and follow-up inputs that may influence how the interaction proceeds.

Control theory offers a coherent framework for explaining why perceived control may shape users’ psychological closeness to AI chatbots. From a control-theoretic perspective, interaction can be understood as an ongoing feedback process in which individuals compare intended reference states with perceived current states and adjust their behavior to reduce discrepancies (Carver and Scheier, 1982). When users perceive that such discrepancy reduction is feasible during interaction, they are more likely to experience predictability and manageability, giving rise to a sense of control over the interaction process (Skinner, 1996; Wu, 2005). Importantly, this control experience does not stem from a single source. In conversational contexts, users may experience control through their ability to regulate the flow and pacing of interaction as well as through their perceived influence over the relevance, focus, and direction of informational content (Ariely, 2000; Benke et al., 2022; Whang et al., 2022). The distinction between process-oriented control and content-oriented control is particularly important for generative AI chatbot interaction because users may feel able to steer how the conversation unfolds without necessarily feeling that they can fully shape what the chatbot ultimately produces. Accordingly, perceived control in conversational AI is better understood as a multifaceted, interaction-proximal experience rather than a unitary or purely outcome-based perception.

Building on these insights, this study develops a control-centered perspective to explain immersive experiences in human–AI chatbot interactions. Extending prior work that has examined psychological distance mainly in relation to AI chatbot design or communication cues, we examine perceived control as an interaction-proximal experience associated with users’ perceived closeness to the AI chatbot. When users feel able to regulate the pacing of the conversation or guide the content toward their goals, the interaction is more likely to be experienced as coherent, responsive, and aligned with their intentions. Under such conditions, the exchange may be perceived as more immediate and less psychologically distant (Carver and Scheier, 1982; Trope et al., 2007). In turn, shorter perceived psychological distance may facilitate users’ focused attention and cognitive involvement, thereby supporting immersion in AI chatbot interaction (Trautmann, 2019; Trope et al., 2007).

Taken together, this study examines how perceived control contributes to immersive AI chatbot interaction through psychological distance. By distinguishing perceived interaction control from perceived content control, we investigate how users’ felt ability to steer the conversational process and shape AI-generated responses is associated with perceived temporal, spatial, and social closeness to AI chatbots. The proposed framework is tested using PLS-SEM and complemented with fsQCA to capture both net effects and alternative configurational pathways to high immersion. In doing so, this study extends psychological distance theory to the context of multi-turn generative AI chatbot interaction, clarifies perceived control as an upstream experiential mechanism by distinguishing users’ perceived control over conversational process from their perceived control over AI-generated content, and provides a more nuanced explanation of immersion by integrating net-effect and configurational perspectives.

## 2 Theoretical background

### 2.1 AI chatbot and perceived control

Unlike traditional one-way information systems, AI chatbots are based on dialogue, making users active participants in the interaction process rather than mere recipients of information. Previous studies have pointed out that AI chatbots rely on users’ continuous input to drive the conversation, with the content, order of information, and interaction pace dynamically adjusted based on user feedback (Alharbi et al., 2025; Brandtzaeg et al., 2022; Chen et al., 2025; Gnewuch et al., 2024). In this sense, users actively engage in generating and shaping the flow of information through ongoing input, corrections, and follow-up questions, rather than passively receiving pre-determined outcomes from the system. This highly interactive communication structure shifts human-computer interaction from a “system-driven information presentation” to a “co-constructed experiential process between the user and the system.”

When users continue to adjust their input, guide the conversation, and observe how the system’s feedback changes accordingly, this cycle of action and feedback gradually transforms into a subjective experience of control. Control is not merely about the objective manipulation of the system’s functionality but is rather a subjective judgment based on whether individuals believe their actions can influence the environment and produce predictable results (Bandura, 1989; Coyle et al., 2007). Based on this understanding, we define perceived control as a subjective sense of agency formed during the dialogue process, where the core lies in whether users believe their inputs can meaningfully influence the direction of the conversation and the final responses generated by the system. This concept emphasizes the manipulability and adjustability of the interaction in the moment, rather than the objective authority or technical operational range that users have over the system. Thus, in the AI chatbot context, control is better understood as a perceived experience rather than a power distribution based on institutional or technical aspects.

This understanding of perceived control is closely related to the interactivity literature, which has often conceptualized perceived interactivity as a multidimensional perception in which user control is one important component, alongside features such as communication direction, responsiveness, and time-related aspects (McMillan and Hwang, 2002; Wu, 2005). However, the present study focuses more specifically on the control-related aspect of interactivity in generative AI chatbot interaction. In this context, users’ perceived control is not simply a global evaluation of whether the system is interactive, but concerns which aspects of the conversational exchange users feel able to influence.

Further, the sense of control formed in AI chatbot interactions is not a single dimension but can be distinguished along the lines of conversational process and generated content. On the one hand, users may feel in control of the rhythm, sequence of information, and the pace of conversation, which reflects process-oriented control over how the interaction unfolds (McMillan and Hwang, 2002; Wu, 2005). On the other hand, users may also form a sense of control based on whether the chatbot’s generated responses can be guided toward their goals, representing content-oriented control over what the interaction produces. Existing research in information control and interaction theory has clearly distinguished between control over the information flow and control over decision outcomes, highlighting the differences in cognitive experience and behavioral responses (Ariely, 2000; Whang et al., 2022). Therefore, in the context of AI chatbots, distinguishing between process-level control and content-level control helps to better understand the different psychological experiences that users form during interaction.

In the present study, this process–content distinction is specified through two constructs: perceived interaction control and perceived content control. Perceived interaction control captures users’ perceived procedural influence over how the conversation unfolds, including pacing, topic shifts, and turn-by-turn progression. Perceived content control captures users’ perceived influence over the substance of AI-generated responses, including the relevance, focus, and direction of the generated responses. This distinction provides a concrete conceptual basis for examining how different forms of perceived control shape psychological distance and subsequently immersion in AI chatbot interaction.

### 2.2 Psychological distance

Psychological distance, grounded in construal level theory, describes how perceived closeness to a target shapes mental representation and experience. When a target feels psychologically close, people tend to rely on concrete, contextualized processing; when it feels distant, they are more likely to adopt abstract and decontextualized construals that emphasize stable and essential features of the target (Trope and Liberman, 2003). This general logic has been widely used to explain differences in cognition, affect, and experiential engagement across contexts.

Psychological distance is typically discussed in terms of temporal, spatial, and social distance, which capture different ways of experiencing separation from an object or interaction partner. Temporal distance concerns how near or far an event feels in time, with temporally proximal targets eliciting more detail-rich, concrete processing (Liberman et al., 2002). In AI chatbot interaction, temporal distance is closely tied to the perceived immediacy and continuity of the dialogue; timely, responsive exchanges can make the interaction feel more “in the moment,” thereby supporting sustained involvement (Meng et al., 2025). Spatial distance reflects perceived separation in space and is not limited to objective physical location, as it can also be shaped by presence cues (Fujita et al., 2006). For AI chatbots, spatial distance is often experienced through the degree of co-presence suggested by the interface and the interaction style; more human-like cues can strengthen a sense of being “with” the system and reduce felt distance (Li and Sung, 2021). Social distance captures perceived relational closeness to the interaction target, with socially proximate targets prompting more personalized, concrete representations (Stephan et al., 2010). In AI chatbot interactions, social distance may be shaped by whether the AI chatbot is experienced as relatable and responsive in ways that invite a sense of interpersonal closeness, which is then associated with stronger emotional engagement.

In the present study, psychological distance is treated as users’ perceived temporal, spatial, and social closeness to the AI chatbot during interaction. This treatment draws on construal level theory, which emphasizes that perceived psychological closeness shapes individuals’ mental representation and involvement (Trope and Liberman, 2003; Trope et al., 2007). It is also consistent with AI chatbot research that has examined psychological distance as a mechanism linking anthropomorphic representations, human-like cues, or conversational strategies to user responses (Li and Sung, 2021; Meng et al., 2025; Park et al., 2024). Building on the perceived control perspective developed above, we focus on users’ perceived influence during the conversation and examine how this control-related experience is associated with perceived temporal, spatial, and social closeness to the AI chatbot, as well as how these dimensions of psychological distance are related to immersion during interaction.

## 3 Research model and hypotheses

### 3.1 Perceived interaction control and psychological distance

Construal level theory holds that psychological distance shapes how concretely people represent and engage with a target (Trope et al., 2007; Trope and Liberman, 2003). In interaction settings, distance is not only a function of objective conditions but can also shift with users’ momentary experience during the exchange. A central experiential factor in conversational interaction is perceived interaction control, namely whether users feel able to steer how the dialogue proceeds (Johnson et al., 2006; McMillan and Hwang, 2002).

When users experience interaction control in AI chatbot conversations, the exchange tends to feel guided and continuous rather than externally paced. Users can adjust their participation across turns, keep the dialogue aligned with their immediate intent, and maintain a sense of continuity as the conversation develops. Under such conditions, the interaction is more likely to be experienced as temporally near, because it is not treated as something deferred or detached from the current moment (Forster et al., 2004). When interaction control is weak, by contrast, the dialogue may feel less continuous and more system-driven, which can weaken the feeling that the exchange is unfolding close to “now” (Lim et al., 2012). Therefore, this study proposes that:

*H1*: Perceived interaction control has a positive effect on reducing temporal psychological distance in AI chatbot interaction.

Spatial psychological distance concerns whether a target is experienced as experientially accessible rather than remote (Fujita et al., 2006). In digital interaction, this form of “closeness” does not depend on physical location but on whether the interaction feels like a shared experiential space that users can enter and navigate (Badrinarayanan et al., 2015; Williams et al., 2005). When users perceive interaction control, they typically experience themselves as participating inside the exchange rather than merely receiving outputs from an external source. This participation-based accessibility makes the AI chatbot feel less distant in spatial terms, because the interaction itself becomes a space the user can actively move within. Therefore, this study proposes that:

*H2*: Perceived interaction control has a positive effect on reducing spatial psychological distance in AI chatbot interaction.

Social psychological distance reflects whether the interaction partner is experienced as relationally close and responsive, or instead as detached and impersonal (Aron, 1991). Importantly, social distance in mediated interaction can arise not only from surface cues, but also from the felt contingency between one’s input and the partner’s responses (Burgoon et al., 2002; Fortin and Dholakia, 2005). In AI chatbot conversations, when users perceive interaction control, their contributions are experienced as shaping the ongoing exchange, and the AI chatbot’s responses appear more contingent on what the user does across turns. This sense of mutual adjustment can make the AI chatbot feel less socially distant, because it is encountered as an interaction counterpart rather than a one-way information source. Therefore, this study proposes that:

*H3*: Perceived interaction control has a positive effect on reducing social psychological distance in AI chatbot interaction.

### 3.2 Perceived interaction control and perceived content control

Perceived interaction control concerns users’ felt ability to steer the procedural flow of conversation, including pacing, turn-by-turn direction, and how the exchange is organized. Perceived content control, by contrast, concerns whether users feel capable of shaping what the AI chatbot ultimately produces in substance, such as the relevance, focus, and usefulness of the generated content. Although conceptually distinct, these experiences are closely linked in multi-turn AI chatbot dialogue.

In practice, users often reach content alignment through process-level steering (Burgoon et al., 2002). When users can redirect the dialogue, clarify constraints, and progressively narrow or refine what they ask for, they gain leverage over what the AI chatbot delivers. The ability to manage the interaction flow therefore provides the conditions under which users can experience content as adjustable in line with their intent. When users feel unable to steer the conversation, they are less likely to experience the output as something they can meaningfully shape, even if the interface technically allows continued input. On this basis, this study proposes the following hypothesis:

*H4*: Perceived interaction control has a positive effect on perceived content control in AI chatbot interaction.

### 3.3 Perceived content control and psychological distance

When users iteratively clarify, refine, and redirect their prompts such that the AI chatbot’s responses increasingly align with their intentions, the interaction is experienced as coherent and goal-oriented. Instead of appearing as isolated replies, the conversation is experienced as an unfolding sequence that stays connected to what the user is trying to achieve.

From a temporal perspective, content-level influence can strengthen the feeling that the exchange is developing in step with the user’s ongoing intent (Johnson et al., 2006; McMillan and Hwang, 2002). When each turn is experienced as moving the output closer to what the user needs, the conversation is less likely to feel deferred or oriented toward an uncertain later result. Rather, it is experienced as temporally near and continuously unfolding, which corresponds to reduced temporal psychological distance. Accordingly, this study proposes the following hypothesis:

*H5*: Perceived content control has a positive effect on reducing temporal psychological distance in AI chatbot interaction.

Content-level influence may also shape spatial distance through perceived accessibility (Fujita et al., 2006). When users experience the AI chatbot’s content as adjustable and responsive to their intent, the system is not encountered as a remote source delivering fixed answers, but as an accessible interaction space in which meaning can be actively shaped. This sense of accessibility can reduce felt separation and make the AI chatbot feel spatially closer within the user’s experiential field. Therefore, this study proposes that:

*H6*: Perceived content control has a positive effect on reducing spatial psychological distance in AI chatbot interaction.

Finally, content control can contribute to social closeness by strengthening perceived responsiveness and alignment. When users perceive that their intentions are being captured and progressively reflected in the AI chatbot’s outputs, the exchange can take on a more relational character (Aron, 1991; Burgoon et al., 2002; Kuo and Feng, 2013). The AI chatbot is experienced less as an impersonal tool and more as a responsive counterpart in the interaction, which can reduce perceived relational separation and strengthen social proximity. Therefore, this study proposes that:

*H7*: Perceived content control has a positive effect on reducing social psychological distance in AI chatbot interaction.

### 3.4 Psychological distance and immersion

Immersion in AI chatbot interaction concerns the extent to which users become absorbed in the unfolding exchange. When temporal psychological distance is reduced, users are less likely to treat the conversation as something detached from the present and more likely to stay engaged with what is unfolding turn by turn (Forster et al., 2004; Kardes et al., 2006; Trope and Liberman, 2003). This temporal closeness supports sustained attention and makes it easier for users to remain involved as the interaction progresses. Therefore, this study proposes that:

*H8*: Reducing temporal psychological distance has a positive effect on immersion in AI chatbot interaction.

When spatial psychological distance is reduced, the AI chatbot is experienced as experientially accessible rather than remote (Fujita et al., 2006). In digital interaction, such spatial closeness often manifests as a felt sense of presence, namely that the interaction target lies within one’s experiential field (Chen and Yen, 2004). When users feel spatially close to the AI chatbot, the conversation can be experienced as an ongoing interaction episode rather than information arriving from an external source, which facilitates absorption and involvement. Prior work also suggests that cues that increase perceived closeness to an AI assistant are associated with more engaged responses (Li and Sung, 2021). Therefore, this study proposes that:

*H9*: Reducing spatial psychological distance has a positive effect on immersion in AI chatbot interaction.

Social psychological distance is closely tied to whether the AI chatbot is experienced as relationally accessible and personally relevant (Stephan et al., 2010). When social distance is reduced, users are more likely to treat the conversation as meaningful beyond a purely instrumental exchange, which encourages greater allocation of cognitive and emotional resources during interaction (Kuo and Feng, 2013). As a result, social proximity is expected to support deeper immersion in AI chatbot dialogue. Therefore, this study proposes that:

*H10*: Reducing social psychological distance has a positive effect on immersion in AI chatbot interaction.

**Figure 1:**
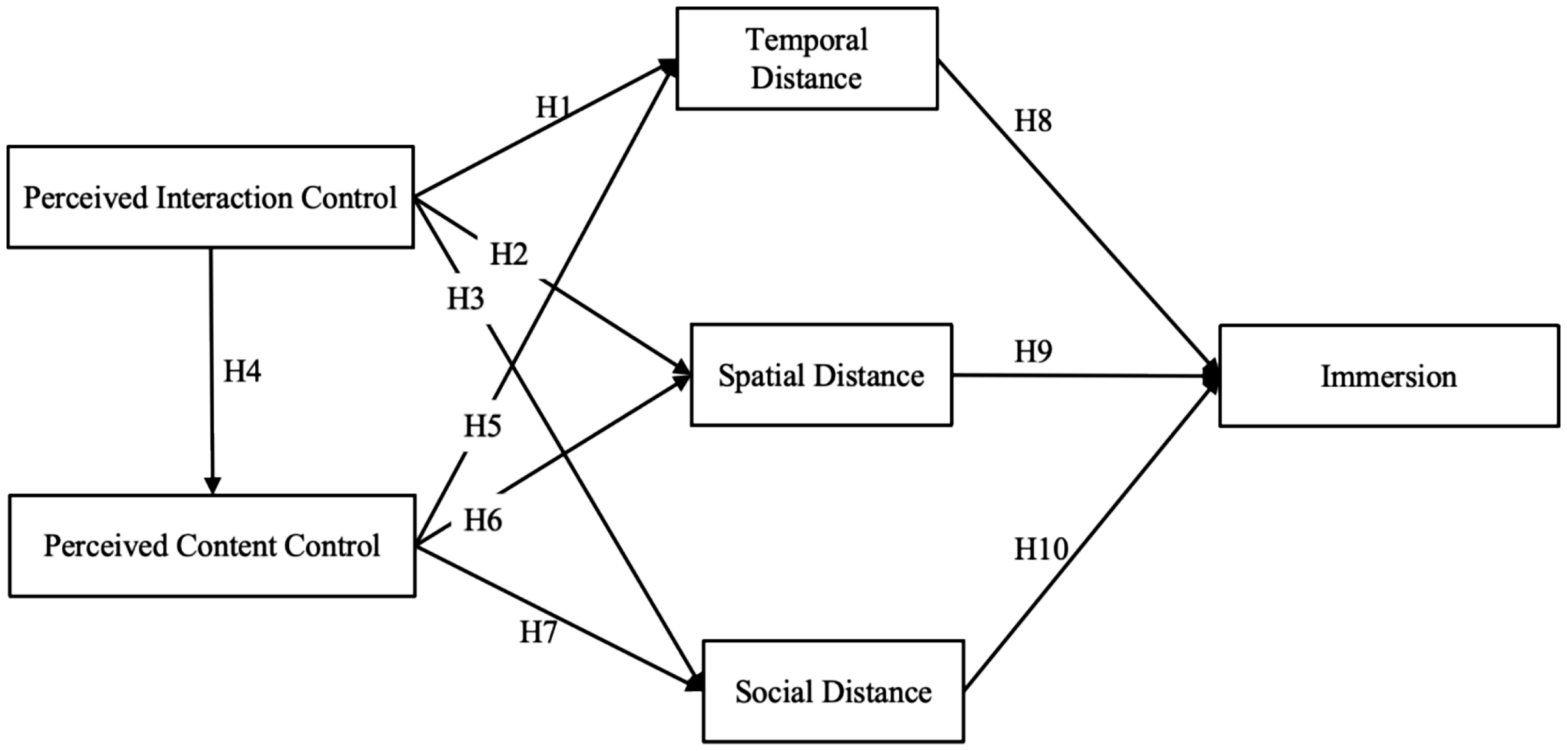
Research model.

Based on the above discussion, we constructed our model in Figure 1.

## 4 Research methodology

### 4.1 Measures and data collection

Measurement items were drawn from established scales where available and adapted to the AI chatbot interaction context. Items measuring psychological distance were adapted from prior studies (Lim et al., 2012) and items were phrased in a proximity-oriented manner; thus, higher scores indicate shorter (i.e., reduced) psychological distance. Immersion was measured using established scales (Yim et al., 2017). Two constructs, perceived interaction control and perceived content control, were developed for this study. These constructs were conceptually grounded in prior research on perceived interactivity and user control in interactive environments (Wu, 2005), but were substantially adapted to reflect the unique characteristics of AI chatbot interaction. Specifically, perceived interaction control items were designed to capture users’ perceived control over the conversational process, including pacing, topic movement, and turn-by-turn progression, whereas perceived content control items were designed to capture users’ perceived control over AI-generated content, including the direction, relevance, and focus of chatbot responses. This item-development logic was aligned with the process–content distinction discussed in the theoretical background, with the aim of capturing two distinct domains of perceived control in AI chatbot interaction.

All items were measured using a five-point Likert-type scale. For agreement-based items, responses ranged from 1 = strongly disagree to 5 = strongly agree. For bipolar immersion items, the endpoints were anchored by the paired descriptions shown in Appendix A. Higher scores indicate higher levels of the corresponding construct. The detailed test items of the research are included in Appendix A. The questionnaire was originally developed in English and then translated into Chinese using a translation and back-translation procedure to ensure semantic equivalence.

Data collection followed a two-stage procedure. A pilot test was first conducted to assess item clarity and the preliminary factor structure of the newly developed control measures. An exploratory factor analysis (EFA) supported the intended two-factor structure, with all items loading on their corresponding constructs (see Appendix B). Based on the pilot results, the items were retained for the formal survey.

The formal survey was administered between May and June 2025 via Credamo, a professional online survey platform widely used in academic research in China. Participation was voluntary and anonymous, and no personally identifiable information was collected. Only respondents who reported having prior experience interacting with AI chatbots were eligible to proceed with the questionnaire. A total of 350 responses were collected. After applying data quality screening criteria, including completion time and attention checks, 338 valid responses were retained for subsequent analysis. The final profile of the samples is listed in Table 1. The proposed model was tested using PLS-SEM and fsQCA to examine cross-sectional relationships and configurational patterns among perceived control, psychological distance, and immersion.

**Table 1:** Sample characteristics.

Demographic variable	Category	Frequency	Percent (%)
Gender	Male	128	37.9
	Female	210	62.1
Age	<18	32	9.5
	18–25	217	64.2
	26–30	39	11.5
	31–35	30	8.9
	>35	20	5.9
Education	High school or below	16	4.7
	Junior college	34	10.1
	Undergraduate	257	76
	Postgraduate or higher	31	9.2

### 4.2 PLS-SEM results

Given that the proposed relationships among the variables have not been sufficiently validated in prior research, this study is exploratory and prediction-oriented, with a focus on theory development. Therefore, the use of PLS-SEM is appropriate (Sarstedt et al., 2022).

#### 4.2.1 Common methods bias

To mitigate common method bias (CMB) potentially arising from single-source survey data, statistical remedies were employed to assess the extent of its influence. First, Harman’s single-factor test showed that the first factor accounted for 19.3% of the total variance, which was below the recommended threshold of 50%, indicating that CMB was unlikely to be a serious concern (Podsakoff et al., 2003). Second, following prior studies, we added a common method factor to the PLS model and linked it to all measurement items of the principal constructs (Liang et al., 2007, 2019). We then calculated the variance of each indicator substantively explained by its principal construct and by the common method factor, respectively. Subsequently, we examined the average variance explained by the substantive constructs and the common method factor. As shown in Appendix C, the ratio of substantive variance to method variance was approximately 246:1, indicating that the substantive variance of the indicators was substantially higher than their method variance (Zhang et al., 2026). In addition, most common method factor loadings were insignificant. Taken together, these results suggest that common method bias was unlikely to pose a serious threat to this study.

#### 4.2.2 Measurement model assessment

The measurement model was assessed in terms of reliability, convergent validity, and discriminant validity. First, internal consistency reliability was evaluated using Cronbach’s alpha (CA) and composite reliability (CR). As reported in Table 2, all CA and CR values exceeded the recommended threshold of 0.70, indicating satisfactory internal consistency reliability. In addition, all item loadings presented in Appendix D exceeded 0.70, providing evidence of adequate indicator reliability. Second, the AVE values for all constructs were above the recommended threshold of 0.50, supporting satisfactory convergent validity. Finally, discriminant validity was tested using both the heterotrait-monotrait ratio of correlations (HTMT) and cross-loadings. As reported in Table 3, all HTMT values were lower than the recommended threshold of 0.85, including the value between perceived interaction control and perceived content control, which was 0.480. Moreover, the cross-loading results shown in Appendix D indicate that each indicator loaded more strongly on its corresponding construct than on any other construct. Taken together, these results indicate that the measurement model satisfies the recommended criteria for discriminant validity.

**Table 2:** Composite reliability, Cronbach’s alphas and AVE.

Construct	CA	CR	AVE
IMM	0.748	0.856	0.666
PCC	0.776	0.870	0.690
PIC	0.830	0.897	0.745
SOD	0.881	0.927	0.809
SPD	0.840	0.903	0.757
TED	0.840	0.903	0.757

**Table 3:** HTMT ratios.

Construct	IMM	PCC	PIC	SOD	SPD	TED
IMM						
PCC	0.703					
PIC	0.610	0.480				
SOD	0.754	0.632	0.345			
SPD	0.654	0.506	0.470	0.616		
TED	0.728	0.581	0.535	0.534	0.706	

#### 4.2.3 Structural model assessment

**Figure 2:**
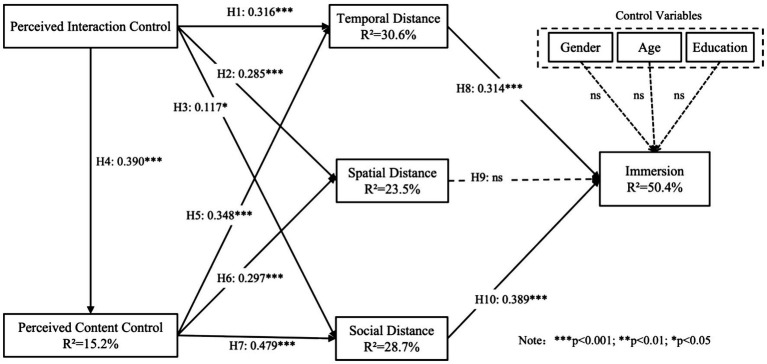
Results of structural model testing.

The structural model was assessed using partial least squares structural equation modeling (PLS-SEM). Figure 2 presents the estimated structural model, and the results of hypothesis testing are summarized in Table 4. The model explained a substantial proportion of variance in immersion (*R*^2^ = 50.4%). In addition, the explained variance was 30.6% for temporal distance, 23.5% for spatial distance, and 28.7% for social distance. 15.2% of the variance in perceived content control was explained. PLSpredict analysis further indicated that all endogenous constructs exhibited positive *Q*^2^ values, suggesting adequate predictive relevance of the proposed model.

**Table 4:** Summary of hypothesis test results.

Hypothesis	Path	*β*	*t*-value	Conclusion
H1	PIC → TED	0.316	5.749	Support***
H2	PIC → SPD	0.285	5.141	Support***
H3	PIC → SOD	0.117	2.100	Support*
H4	PIC → PCC	0.390	8.125	Support***
H5	PCC → TED	0.348	6.792	Support***
H6	PCC → SPD	0.297	5.273	Support***
H7	PCC → SOD	0.479	9.116	Support***
H8	TED → IMM	0.314	5.615	Support***
H9	SPD → IMM	0.116	1.847	No Support
H10	SOD → IMM	0.389	6.717	Support***

***p < 0.001; **p < 0.01; *p < 0.05.

Regarding the proposed relationships, perceived interaction control showed significant positive effects on reduced temporal distance (H1: *β* = 0.316, *p* < 0.001), spatial distance (H2: *β* = 0.285, p < 0.001), and social distance (H3: *β* = 0.117, *p* < 0.05). Perceived interaction control was also positively associated with perceived content control (H4: *β* = 0.390, *p* < 0.001). These results provide support for H1, H2, and H4, and indicate a weaker but statistically significant effect for H3.

Furthermore, perceived content control exhibited significant positive effects on reduced temporal distance (H5: *β* = 0.348, *p* < 0.001), spatial distance (H6: *β* = 0.297, *p* < 0.001), and social distance (H7: *β* = 0.479, *p* < 0.001), thereby supporting H5, H6, and H7.

With respect to the outcome variable, reduction of temporal distance (H8: *β* = 0.314, *p* < 0.001) and social distance (H10: *β* = 0.389, *p* < 0.001) were positively associated with immersion. However, the effect of spatial distance on immersion was not significant (H9). Thus, H8 and H10 were supported, whereas H9 was not supported. Control variables, including gender, age, and education, showed no significant effects on immersion.

### 4.3 fsQCA results

#### 4.3.1 Calibration and necessity analysis

In addition to PLS-SEM, this study employs fuzzy-set qualitative comparative analysis (fsQCA) to further examine the relationships among perceived control, psychological distance, and immersion. While PLS-SEM focuses on estimating net effects and average linear relationships, fsQCA adopts a configurational perspective to capture configurational complexity and explore how different combinations of conditions are associated with high immersion. This approach is particularly suitable for AI chatbot emotional interaction contexts, where user experiences may emerge through multiple, non-symmetric pathways rather than a single dominant mechanism (Fiss, 2007; Ragin, 2008).

Before conducting the configurational analysis, all variables were calibrated into fuzzy-set membership scores using the direct calibration method. Given that the variables were measured using five-point Likert-type scales and that the response distributions differed across constructs, this study adopted a percentile-based direct calibration strategy. Specifically, the 95th, 50th, and 5th percentiles of each variable were used as the anchors for full membership, the crossover point, and full non-membership, respectively. The crossover point therefore represents the empirical point at which cases are neither more in nor more out of a given set. This calibration strategy allows the fuzzy-set membership scores to reflect the distribution of respondents’ perceptions while preserving meaningful distinctions between relatively high, ambiguous, and low membership in each condition (Prentice et al., 2021; Woodside, 2014). Table 5 reports the calibration thresholds for all conditions and the outcome variable used in the fsQCA analysis.

**Table 5:** Calibration anchors for fsQCA.

Variable	Calibrations (0.95, 0.5, 0.05)
PIC	4.6667, 3.6667, 2.6667
PCC	4.3333, 3, 1.6667
TED	4.6667, 3.3667, 2.3333
SPD	4.6667, 3.3333, 1.6167
SOD	4.3333, 3,1
IMM	4.6667, 3.3333, 1.6667

Following calibration, a necessity analysis was performed to examine whether any single condition consistently appeared in cases of high immersion. As shown in Table 6, none of the examined conditions reached the consistency threshold required to be considered a necessary condition. This suggests that high immersion in AI chatbot interactions does not depend on any single factor alone, but instead is associated with specific combinations of perceived control and psychological distance conditions.

**Table 6:** Results for necessary conditions testing.

Conditions	Consistency	Coverage
PIC	0.837970	0.751296
~PIC	0.468090	0.593358
PCC	0.752431	0.803406
~PCC	0.552220	0.570655
TED	0.799823	0.793283
~TED	0.520513	0.580927
SPD	0.804313	0.804313
~SPD	0.519944	0.583446
SOD	0.768257	0.824779
~SOD	0.527713	0.542481

Outcome variable: Immersion.

#### 4.3.2 Truth table and solution analysis

Based on the calibrated data, a truth table analysis was conducted to identify sufficient configurations associated with high immersion. Following established fsQCA procedures, a frequency threshold of 5 cases and a consistency threshold of 0.90 were applied. The resulting intermediate solution yielded five distinct configurational paths associated with high immersion, as summarized in Table 7.

**Table 7:** Five sufficient configurations for high Immersion.

Configuration	Solution for high immersion				
	S1	S2	S3	S4	S5
PIC	●	●	●	●	⊗
PCC	●	●	●		●
TED	●		●	●	⊗
SPD	●	●		●	⊗
SOD		●	●	●	●
Raw coverage	0.556045	0.538011	0.538158	0.562581	0.25518
Unique coverage	0.0471047	0.0181974	0.0207214	0.0536402	0.0335103
Consistency	0.941783	0.959354	0.955573	0.959121	0.910194
Solution coverage	0.701484				
Solution consistency	0.906234				

Large black circles indicate core presence; small black circles indicate peripheral presence; large crossed circles indicate core absence; small crossed circles indicate peripheral absence; blank cells indicate that the condition is irrelevant or “do not care” in the corresponding configuration.

As shown in Table 7, no single condition was sufficient on its own to produce high immersion. Instead, high immersion was associated with multiple combinations of perceived control and psychological distance conditions. Across the five configurations, perceived content control and reduced social distance appeared as recurring core conditions, while perceived interaction control, temporal distance, and spatial distance played different roles across configurations. The overall solution exhibited high consistency (0.906) and substantial coverage (0.701), indicating that the identified configurations reliably explain a large proportion of high-immersion cases.

To assess robustness, additional analyses were conducted by varying the frequency threshold and consistency cutoff. The resulting solutions remained substantively similar to the main solution, indicating that the configurational findings are robust to alternative parameter settings.

## 5 Discussion

This study examined how perceived control shapes immersion in AI chatbot interactions by linking users’ control experience to multiple facets of psychological distance. By combining PLS-SEM and fsQCA, the findings offer convergent evidence from both net-effect estimation and configurational analysis regarding how immersive engagement emerges during AI chatbot-based exchanges.

### 5.1 The role of perceived control in reducing psychological distance

The PLS-SEM results underscore the importance of perceived control in shaping users’ psychological distance to the AI chatbot. Perceived interaction control showed significant positive effects on the reduction of temporal, spatial, and social psychological distance, suggesting that when users feel able to steer how the conversation unfolds, the interaction is experienced as more immediate, less experientially remote, and less relationally detached. These findings align with prior research suggesting that psychological distance in mediated interaction is shaped not only by structural cues but also by users’ experiential engagement during exchange (Lim et al., 2012), and they are consistent with foundational views of perceived interactivity emphasizing user control and time-based responsiveness as core experiential components (McMillan and Hwang, 2002). Perceived interaction control also exhibited a strong positive effect on perceived content control, indicating that users’ procedural influence over the dialogue tends to translate into a stronger sense of influence over what the AI chatbot ultimately produces. Taken together, these results suggest that interaction-level control and content-level control form closely connected components of users’ overall control experience in AI chatbot conversations.

Perceived content control further demonstrated consistent positive effects on the reduction of temporal, spatial, and social psychological distance. When users felt able to refine, redirect, and shape the substantive focus of the AI chatbot’s output, the exchange was more likely to be experienced as ongoing and coherent, with a stronger sense of immediacy and relational accessibility. In this sense, content-level influence appears to contribute directly to shortening psychological distance across multiple dimensions, reinforcing the view that the perceived ability to shape AI-generated content is closely tied to how “near” and responsive the interaction feels from the user’s perspective (Burgoon et al., 2002; Fortin and Dholakia, 2005).

### 5.2 The differentiated effects of psychological distance on immersion

With respect to immersion, the SEM results reveal differentiated effects across dimensions of psychological distance. Reductions in temporal and social psychological distance were both positively related to immersion, whereas the effect of reduced spatial psychological distance was not significant. This pattern suggests that, in the present AI chatbot context, immersive engagement is more strongly driven by the felt “here-and-now” quality of the exchange and by relational closeness than by spatial experience. One plausible interpretation is that spatial distance becomes more consequential when the interaction interface provides richer co-presence cues, such as voice, embodiment, visual representation, or multimodal feedback. Recent studies on virtual assistants and AI chatbots suggest that embodied or multimodal interaction can strengthen users’ sense of presence, emotional engagement, and immersion by offering richer social and perceptual cues than text-only interaction (de Melo et al., 2020; Peng et al., 2026). By contrast, in text-based conversational AI, users primarily encounter the chatbot through linguistic responses rather than bodily or spatial cues. Thus, the unsupported H9 does not imply that spatial psychological distance is irrelevant to AI interaction in general; rather, it suggests that spatial proximity may be a modality-dependent pathway to immersion, becoming more salient in voice-based, embodied, or immersive AI systems than in purely text-based chatbot interaction.

### 5.3 Configurational pathways to high immersion

The fsQCA findings complement the net-effect results by showing that high immersion can arise through multiple configurational pathways rather than a single uniform route. No single condition reached the threshold for necessity, indicating that immersive engagement does not depend on any one factor alone. Instead, five sufficient configurations were identified, suggesting that different combinations of perceived control and psychological distance are associated with high immersion in AI chatbot interaction.

A closer interpretation of the core and peripheral conditions further clarifies why some experiential factors play more central roles in configurations associated with high immersion. Perceived content control frequently emerged as a core condition, suggesting that immersion in generative AI chatbot interaction is strongly anchored in users’ perceived ability to shape the substantive direction, relevance, and usefulness of the chatbot’s responses. In this context, users are not immersed simply because they can continue a dialogue, but because they feel that the generated content can be progressively adjusted toward their own intentions, which aligns with recent work emphasizing responsiveness and interactional flexibility as important bases of emotional engagement in conversational AI (Lan et al., 2026). Reduced social psychological distance also appeared as a recurring core condition, indicating that high immersion depends on the chatbot being experienced not merely as a functional tool but as a socially responsive and relationally accessible interaction counterpart. This interpretation is consistent with evidence that users’ acceptance of chatbot support is shaped by mind perception and socially meaningful responses (Lee and Hahn, 2024). By contrast, perceived interaction control tended to play a more peripheral or route-specific role. This may be because process-level control supports immersion mainly by enabling users to steer the dialogue and refine the generated content, rather than by serving as the primary basis of immersion in all configurations. Similarly, reduced temporal and spatial psychological distance contributed to high immersion in some pathways, but their roles were more contingent. Together, these patterns indicate that high immersion is most consistently supported by content alignment and social closeness, while other experiential factors operate as pathway-specific enabling conditions.

## 6 Implications and limitations

### 6.1 Theoretical implications

This study contributes to the literature on AI chatbot interaction and user experience in three ways. First, it extends psychological distance theory to the context of multi-turn generative AI chatbot interaction. Prior work in AI contexts has frequently examined psychological distance in relation to interface or communication cues, such as anthropomorphic representation, human-like representations, or predefined response strategies. This study extends this line of work by examining perceived control as an interaction-proximal experience associated with users’ perceived temporal, spatial, and social closeness to AI chatbots. Rather than proposing a new conceptualization of psychological distance, this study applies an established psychological distance perspective to a theoretically underexplored context. This perspective helps explain how users’ perceived closeness to AI chatbots is related to their control experiences in generative conversational settings and shifts attention from externally designed interaction cues alone to users’ perceived influence within the conversational exchange.

Second, the study advances theorizing on user agency by distinguishing between perceived interaction control and perceived content control and clarifying their relationship in AI chatbot conversations. Much of the perceived control literature either treats control as a unitary experience or discusses agency at a broad level in relation to system autonomy (Bennett et al., 2023; Frischknecht, 2021). The present results indicate that users differentiate control over how the conversation proceeds from control over what the AI chatbot ultimately produces, and that interaction-level control provides a basis for content-level control. This distinction deepens the understanding of user agency in conversational AI because users may feel able to steer the flow, pacing, or topic progression of a conversation without necessarily feeling that they can fully shape the relevance, focus, or direction of AI-generated responses. By distinguishing users’ perceived control over the conversational process from their perceived control over generated content, this study provides a more fine-grained and user-centered account of how agency is experienced in generative AI chatbot interaction.

Third, by integrating PLS-SEM with fsQCA, the study offers a complementary theoretical and analytical lens for explaining immersive experiences in human–AI interaction. While PLS-SEM captures average net-effect relationships among perceived control, psychological distance, and immersion, fsQCA identifies multiple sufficient configurations associated with high immersion. This dual perspective contributes to the literature on immersion by showing that immersive AI chatbot experiences are not adequately captured by a single linear explanation. Instead, high immersion can be better understood through both average net-effect relationships and configurational patterns involving different combinations of perceived control and psychological closeness. In particular, the configurational findings suggest that perceived content control and social closeness appear as recurrent conditions across several pathways, whereas interaction control, temporal closeness, and spatial closeness play more pathway-specific roles. These findings provide a more nuanced explanation of immersion in AI chatbot interaction by showing that high immersion is associated with different combinations of experiential conditions rather than a single uniform pathway.

### 6.2 Practical implications

The findings offer several design implications for deploying AI chatbots in emotionally oriented interaction contexts such as companionship and support. First, the results suggest that fostering users’ perceived control should be treated as a core design objective. Beyond improving model capability or adding humanlike surface features, designers can focus on whether users experience the dialogue as steerable and revisable. Interaction patterns that make user input consequential, for instance by enabling easy topic redirection, allowing users to adjust pacing, or supporting iterative refinement of the AI chatbot’s responses, can strengthen the sense that the conversation is developing under the user’s guidance, which is conducive to deeper engagement.

Second, the differentiated roles of psychological distance imply that design resources may be better allocated toward cues that enhance immediacy and relational accessibility rather than toward attempts to simulate physical co-presence. In text-based AI chatbot settings, immersion appears to benefit more from the experience of continuity and “being in the moment,” as well as from signals that the AI chatbot is responsive and relationally approachable. Accordingly, features that support timely follow-ups, coherent multi-turn progression, and a stable interpersonal tone may be more effective for immersion than interface elements aimed primarily at producing a sense of spatial proximity.

Finally, the configurational evidence indicates that there is no single optimal pathway to high immersion. Users can arrive at immersive experiences through different combinations of control and distance perceptions, which suggests the value of flexible interaction design. Rather than enforcing one uniform conversational logic, AI chatbot systems may benefit from offering configurable modes and personalization options that accommodate heterogeneous user preferences, including differences in how much guidance, structure, or conversational autonomy users’ desire.

### 6.3 Limitations

This study has several limitations. First, the use of a cross-sectional survey design limits strong causal inference and does not allow us to directly observe how perceived control, psychological distance, and immersion change across conversational turns or repeated AI chatbot interactions. Although this study treats psychological distance as sensitive to users’ interaction experiences, the empirical design captures users’ perceptions at a single point in time. Therefore, the findings should be interpreted as evidence of relationships among perceived control, psychological distance, and immersion, rather than as direct evidence of within-person changes in psychological distance over time. Future research could employ longitudinal, experimental, or experience-sampling designs to better examine how these processes unfold during multi-turn AI chatbot interactions.

Second, all focal constructs were measured through self-reports. Although appropriate for subjective perceptions such as control and immersion, this approach may introduce common method concerns. Subsequent studies could incorporate behavioral indicators or interaction log data to complement perceptual measures.

Third, the sample primarily consisted of relatively young users with prior AI experience, which may constrain generalizability. Future research could test the proposed model across different age groups, cultural contexts, and AI application domains to further assess its robustness.

## Data Availability

The raw data supporting the conclusions of this article will be made available by the authors, without undue reservation.
