# Supplementary material for: Perceived control and immersion in AI chatbot interaction: a psychological distance perspective
Source: Front Psychol. 2026 Jul 14;17:1823399. doi: 10.3389/fpsyg.2026.1823399 (PMC13407650; doi:10.3389/fpsyg.2026.1823399)
Supplement: Supplementary file 1 [file Supplementary_file_1.docx]

**Appendix A**

| Construct | Item | Measurement Item (English translation) |
| --- | --- | --- |
| Immersion (IMM) | IMM 1 | Not deeply engrossed-Deeply engrossed |
|  | IMM 2 | Not absorbed-Absorbed |
|  | IMM 3 | My attention was not focused-My attention was focused |
| Perceived Interaction Control (PIC) | PIC 1 | I can regulate the pacing of the conversation with the AI chatbot. |
|  | PIC 2 | When I shift to a new topic, the AI chatbot follows my lead smoothly. |
|  | PIC 3 | The AI chatbot follows my lead during the conversation. |
| Social distance  (SOD) | SOD 1 | It was easy for me to feel socially connected to the AI chatbot during the interaction. |
|  | SOD 2 | I felt a sense of relational closeness with the AI chatbot during the interaction. |
|  | SOD 3 | The interaction with the AI chatbot felt socially meaningful to me. |
| Spatial distance  (SPD) | SPD 1 | I felt I was in the same place as the AI chatbot during the interaction. |
|  | SPD 2 | I felt that I was interacting with the AI chatbot within the same shared interaction space. |
|  | SPD 3 | I felt the interaction with the AI chatbot took place in the same space as I was in. |
| Temporal distance  (TED) | TED 1 | I felt I was interacting with the AI chatbot simultaneously during the interaction. |
|  | TED 2 | I felt that the interaction with the AI chatbot unfolded in real time. |
|  | TED 3 | The AI chatbot responded quickly to my inputs during the interaction. |
| Perceived Content Control (PCC) | PCC 1 | I can guide the direction of the AI chatbot’s responses during the interaction. |
|  | PCC 2 | I can adjust the content focus of the AI chatbot’s replies to better match my intentions. |
|  | PCC 3 | I can modify the AI chatbot’s responses by refining or redirecting my input. |

**Appendix B**

| Item | Perceived Interaction Control  (PIC) | Perceived Content Control (PCC) |
| --- | --- | --- |
| PIC1 | 0.824 |  |
| PIC2 | 0.795 |  |
| PIC3 | 0.811 |  |
| PCC 1 |  | 0.775 |
| PCC 2 |  | 0.824 |
| PCC 3 |  | 0.784 |

**Appendix C**

| Construct | Indicator | Substantive factor loading(R1) | R1^2^ | Method factor loading(R2) | R2^2^ |
| --- | --- | --- | --- | --- | --- |
| IMM | IMM1 | 0.800*** | 0.641 | 0.113 | 0.013 |
|  | IMM2 | 0.853*** | 0.728 | -0.022 | 0.000 |
|  | IMM3 | 0.793*** | 0.628 | -0.092 | 0.009 |
| PCC | PCC1 | 0.862*** | 0.743 | 0.015 | 0.000 |
|  | PCC2 | 0.809*** | 0.654 | -0.051 | 0.003 |
|  | PCC3 | 0.821*** | 0.675 | 0.033 | 0.001 |
| PIC | PIC1 | 0.853*** | 0.728 | -0.043 | 0.002 |
|  | PIC2 | 0.881*** | 0.776 | 0.115*** | 0.013 |
|  | PIC3 | 0.857*** | 0.734 | -0.081* | 0.007 |
| SOD | SOD1 | 0.906*** | 0.821 | -0.035 | 0.001 |
|  | SOD2 | 0.916*** | 0.839 | -0.010 | 0.000 |
|  | SOD3 | 0.875*** | 0.766 | 0.046 | 0.002 |
| SPD | SPD1 | 0.874*** | 0.765 | -0.025 | 0.001 |
|  | SPD2 | 0.876*** | 0.768 | 0.026 | 0.001 |
|  | SPD3 | 0.860*** | 0.739 | -0.001 | 0.000 |
| TED | TED1 | 0.871*** | 0.758 | -0.014 | 0.000 |
|  | TED2 | 0.870*** | 0.757 | 0.003 | 0.000 |
|  | TED3 | 0.870*** | 0.756 | 0.011 | 0.000 |
| AVG |  |  | 0.738 |  | 0.003 |

**Appendix D**

|  | IMM | PCC | PIC | SOD | SPD | TED |
| --- | --- | --- | --- | --- | --- | --- |
| **IMM1** | ***0.809*** | 0.465 | 0.430 | 0.498 | 0.418 | 0.534 |
| **IMM2** | ***0.848*** | 0.462 | 0.433 | 0.536 | 0.455 | 0.422 |
| **IMM3** | ***0.789*** | 0.386 | 0.330 | 0.465 | 0.395 | 0.457 |
| **PCC1** | 0.475 | ***0.864*** | 0.351 | 0.451 | 0.356 | 0.407 |
| **PCC2** | 0.412 | ***0.800*** | 0.277 | 0.389 | 0.346 | 0.354 |
| **PCC3** | 0.452 | ***0.828*** | 0.338 | 0.463 | 0.316 | 0.409 |
| **PIC1** | 0.395 | 0.334 | ***0.845*** | 0.250 | 0.310 | 0.322 |
| **PIC2** | 0.489 | 0.367 | ***0.898*** | 0.334 | 0.422 | 0.444 |
| **PIC3** | 0.366 | 0.301 | ***0.845*** | 0.181 | 0.286 | 0.390 |
| **SOD1** | 0.543 | 0.452 | 0.284 | ***0.903*** | 0.462 | 0.399 |
| **SOD2** | 0.551 | 0.503 | 0.270 | ***0.916*** | 0.459 | 0.425 |
| **SOD3** | 0.559 | 0.458 | 0.265 | ***0.878*** | 0.507 | 0.416 |
| **SPD1** | 0.451 | 0.361 | 0.314 | 0.455 | ***0.872*** | 0.512 |
| **SPD2** | 0.446 | 0.346 | 0.378 | 0.475 | ***0.876*** | 0.546 |
| **SPD3** | 0.457 | 0.357 | 0.353 | 0.451 | ***0.863*** | 0.490 |
| **TED1** | 0.481 | 0.418 | 0.419 | 0.368 | 0.520 | ***0.870*** |
| **TED2** | 0.518 | 0.439 | 0.347 | 0.419 | 0.500 | ***0.871*** |
| **TED3** | 0.510 | 0.371 | 0.412 | 0.413 | 0.528 | ***0.869*** |
